# Alumina/nano-graphite composite as a new nanosorbent for the selective adsorption, preconcentration, and determination of chromium in water samples by EDXRF

**DOI:** 10.1007/s00216-018-1397-8

**Published:** 2018-10-05

**Authors:** Anna Baranik, Rafal Sitko, Anna Gagor, Beata Zawisza

**Affiliations:** 1 Institute of Chemistry, University of Silesia, Szkolna 9, 40-006 Katowice, Poland; 2 Institute of Low Temperature and Structure Research, Polish Academy of Sciences, P.O. Box 1410, 50-950 Wrocław, Poland

**Keywords:** Preconcentration, Speciation, Trace analysis, Nanosorbent, Environmental samples, Sorption

## Abstract

Obtaining new nanocomposites with sorption properties towards chromium is highly important not only from the environmental point of view but also for developing eco-friendly methods of chromium determination. The potential use of aluminum oxide-coated nano-graphite (Al_2_O_3_/nano-G) as a new nanosorbent in ultrasound-assisted dispersive micro-solid-phase extraction (DMSPE) for rapid speciation of trace chromium(III) and chromium(VI) ions in natural water was evaluated. In the developed method, the crucial issue is the new nanocomposite synthesized by coating alumina on a nano-graphite surface with sorption properties. Structural researches of the nanocomposite were carried out by scanning electron microscopy (SEM), powder X-ray diffraction (XRD), and Raman spectroscopy. Maximum adsorption capacity of Al_2_O_3_/nano-G towards Cr(III) was 32.8 mg g^−1^. The influence of the method’s factors like pH, sample volumes, contact time, coexisting ions, and humic acid on the recovery of chromium was examined. The nanocomposites have been found to be stable and effective as a sorbent in water with high concentrations of selected cations and anions present in water as well as in water of various pH. Al_2_O_3_/nano-G is selective for Cr(III) in presence of Cr(VI). Cr(III) was determined by the developed method, total Cr after reduction of Cr(VI) to Cr(III), and Cr(VI) was calculated as the difference between total Cr and Cr(III). After sorption, the nanocomposite with chromium was collected on 5-mm diameter filters and analyzed by energy-dispersive X-ray fluorescence spectrometry (EDXRF) to determine the chromium concentration. The method was characterized by correlation coefficient 0.999, limit of detection (LOD) 0.04 ng mL^−1^, and relative standard deviation (RSD) 3.5%. Al_2_O_3_/nano-G combined with proposed DMSPE/EDXRF was verified by analysis of certificate reference material of natural water (NIST 1640a).

**Graphical abstract:**
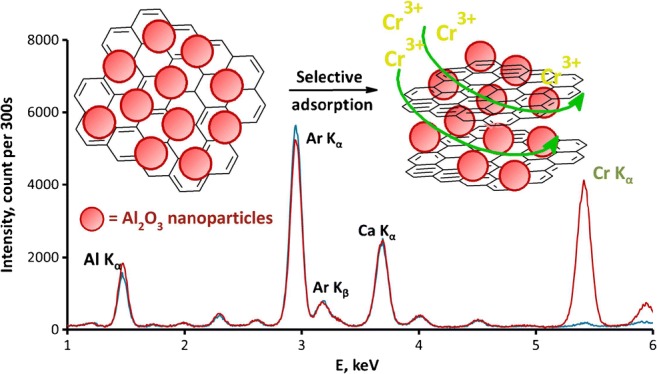
ᅟ

ᅟ

## Introduction

The interest in chromium speciation originates from widespread use of this metal in various industries such as chemical, metallurgical, and refractories. Thus, large quantities of chromium compounds are discharged in solid, liquid, and gaseous wastes into the environment and can have potentially adverse biological and ecological effects. The two widespread oxidation states of Cr are present in the environment, i.e., Cr(III) and Cr(VI). They are drastically different in chemical and biochemical reactivity and physicochemical properties [1]. Cr(III) is considered to be a trace element essential for the proper functioning living organisms, but in a significant concentration can cause adverse effects because of its high capability to coordinate various organic compounds, whereas Cr(VI) is considered exerting toxic effects on biological systems. It was found that exposure to hexavalent Cr compounds can lead to a variety of clinical problems [2]. Thus, a detailed knowledge of each species rather than the total chromium level is required to properly evaluate its toxicological effects, and distribution in the environment. The concentration of chromium in environment is controlled and observed by the World Health Organization (WHO) [3] and the Environmental Protection Agency (EPA) [4]. WHO and EPA have established an upper concentration limit for chromium in drinking water, which equals 50 ng mL^−1^ and 100 ng mL^−1^, respectively.

The literature proposes a lot of methods to oxidation and/or reduction of chromium before the removal/separation. The Cr(III) ions can be oxidized by potassium permanganate solution (KMnO_4_) [5, 6], hydrogen peroxide (H_2_O_2_) [7, 8], or potassium peroxodisulfate (K_2_S_2_O_8_) in acid solution [9]. Many methods allowing reducing Cr(VI) ions are also shown in the literature. The concentrated sulfuric acid (H_2_SO_4_) with ethanol (C_2_H_5_OH) [10, 11], hydroxylamine hydrochloride (NH_2_OH · HCl) solution [12–14], or hydrochloric acid (HCl) solution with C_2_H_5_OH [15] has played a good role in complete reduction of Cr(VI).

For determination of trace amounts of chromium in different materials, oxidized multiwalled carbon nanotubes (MWCNTs) [14], MWCNTs modified with the anionic exchanger tricaprylmethylammonium chloride (Aliquat 336) [16], graphene oxide (GO) [11], graphene oxide decorated with triethylenetetramine-modified magnetite (mf-GO) [17], and graphene (G) [18] were applied. The trace amount of chromium ions was preconcentrated using solid-phase extraction (SPE) [18] and dispersive magnetic SPE [17] combined with flame atomic absorption spectrometry (FAAS), dispersive micro-solid-phase extraction (DMSPE) combined with total reflection X-ray fluorescence (TXRF) [16], and energy-dispersive X-ray fluorescence spectrometry (EDXRF) [11]. Several carbon sorbents have also high affinity for both chromium and other heavy metals. Oxidized MWCNTs [19], 3-(2-aminoethylamino) propyltrimethoxysilane (AAPTS) functionalized MWCNTs [6], Schiff base-chitosan-grafted MWCNTs (S-CS-MWCNTs) [20], nano-graphite [21], GO nanosheets [22], and GO connected with 2-(5-bromo-2-pyridylazo)-5-diethylaminophenol (5-Br-PADAP) [23] have found analytical application in the sorption and determination not only of Cr(III) and Cr(VI) but also of Mn(II), Fe(III), Co(II), Ni(II), Cu(II) Pb(II), As(V), Se(VI), and V(V). Our previous studies have shown that alumina supported on graphene oxide (Al_2_O_3_/GO) can be successfully used for preconcentration of As(V) and Cr(III) [24].

In this work, the alumina was supported on nano-graphite to obtain Al_2_O_3_/nano-G composite suitable for selective sorption of chromium in both batch and under flow conditions. Thanks to microscopy and spectroscopy researches, Al_2_O_3_/nano-G composite structure was confirmed and characterized. The maximum adsorption capacity of the new nanosorbent was evaluated by Langmuir isotherm model. Al_2_O_3_/nano-G composite was applied in DMSPE combined with EDXRF. The advantage of this methodology over ICP-OES, ICP-MS, and FAAS is the possibility of direct determination of analyte onto sorbent without elution. Thus, errors connected with contamination of sample or losses of analyte are eliminated and time of sample preparation is shortened. The preconcentration of Cr(III) by Al_2_O_3_/nano-G can be performed within 5 min. The obtained samples are durable; thus, they can be stored and analyzed many times as well as can be used in further research. Analytes can be also determined after elution from nanocomposite, using alternative techniques. Application of Al_2_O_3_/nano-G in DMSPE/EDXRF method allows for direct determination of trace amounts of Cr(III) in environment water samples. The developed methodology is environmentally friendly and non-time-consuming.

## Materials and methods

### Materials

Chromium stock solution 1 mg mL^−1^ of Cr(III) and Cr(VI) was purchased from Merck (Darmstadt, Germany); humic acid was purchased from Sigma-Aldrich (Steinheim, Germany); graphene nanopowder 8 nm (purity 99.99% and the flakes size 8 nm) was purchased from graphene supermarket (New York, USA); nitric acid (65%, Suprapur®), ammonium hydroxide solution (25%, Suprapur®), ethanol (96% p.a.), sulfuric acid (96% p.a.), Triton X-100 (p.a.), chromium(III) nitrate nanohydrate (p.a.), sodium nitrate (p.a.), potassium nitrate (p.a.), calcium nitrate tetrahydrate (p.a.), magnesium nitrate hexahydrate (p.a.), iron(III) nitrate nanohydrate (p.a.), aluminum nitrate nanohydrate (p.a.), buffer solution (pH 4.00 ± 0.05 and pH 7.00 ± 0.05), and the Munktell membrane filters of thickness 176 μm (no. 391) were purchased from Avantor Performance Materials Poland S.A. (Gliwice, Poland). Standard solutions were diluted with high-purity water obtained from Milli-Q system (Millipore, Molsheim, France). Certified Reference Material (natural water 1640a) was purchased from National Institute of Standards and Technology (Gaithersburg, USA).

### Apparatus

The microstructural observation of the Al_2_O_3_/nano-G was on a JEOL-7600F scanning electron microscope (SEM) (Oregon, USA) equipped with the Oxford X-ray energy-dispersive spectrometer (EDS). Powder X-ray diffraction data (XRD) (PANalytical, Almelo, The Netherlands) were collected on X’Pert PRO X-ray diffractometer with PIXcel ultrafast line detector and Soller slits for Cu K_α_ radiation. The measurements were done in Bragg-Brentano geometry.

The Raman spectra (Renishaw, New Mills, Wotton-under-Edge Gloucestershire, UK) were measured at room temperature using RenishawInVia Raman spectrometer equipped with confocal DM 2500 Leica optical microscope, a thermoelectrically cooled Ren Cam CCD detector and a diode laser operating at 830 nm.

The EDXRF spectra were measured by energy-dispersive X-ray fluorescence spectrometer Epsilon 3 (PANalytical, Almelo, The Netherlands) with the Rh target X-ray tube with 50-μm Be window and max. power of 9 W. The spectrometer is equipped with thermoelectrically cooled silicon drift detector (SDD) with 8-μm Be window and resolution of 135 eV at 5.9 keV. The spectrometer is equipped with spinner and five primary filters that can be selected to improve measuring conditions for determined elements. The measurement conditions are 20 kV, 450 μA, Al filter of 200-μm thickness, air atmosphere, and 300 s of measure time. The samples were packed in mylar foil of 6-μm thickness.

ICP-OES (Spectro Analytical Instruments GmbH, Kleve, Germany) measurements were performed using a SpectroBlue FMS16 spectrometer with inductively coupled plasma (ICP) excitation (Spectro Analytical Instruments) and a charge-coupled-device detector. The following operation parameters were used for measurements: plasma power—1.45 kW; coolant gas—Ar, 12 L min^−1^; auxiliary gas—Ar, 1 L min^−1^; nebulizer gas—Ar, 1 L min^−1^; nebulizer pressure—3.2 bar; nebulizer-cross-flow type; sample uptake rate—2 mL min^−1^; wavelength—267.716 nm for Cr.

Ultrasonication bath with heating, EMAG, Emmi 20 HC model was purchased from EMAG Poland (Juszczyn, Poland); ultrasonic power, 150 W; working range of ultrasonic power, 50–100%; heating power, 200 W; working range of temperature, 20–80 °C; frequency, 45 kHz; and maximum volume, 1.4 L.

### Synthesis of Al_2_O_3_/nano-G composite

Al_2_O_3_/nano-G composite was synthesized as follows: 5 g of Al(NO_3_)_3_ · 9H_2_O, 300 mg of Triton X-100, and 1 g of high-purity graphene nanosheets were sonificated in 100 mL of water for 2 h. The black suspension was dried at 80 °C for 10 h and then heated at 500 °C for 2 h to obtained Al_2_O_3_ nanoparticles supported on nano-graphite (Al_2_O_3_/nano-G) [25].

### Preparation of Al_2_O_3_/nano-G suspension

Two hundred fifty milligrams of Al_2_O_3_/nano-G was placed in 50-mL flasks and filled with high-purity water up to the mark. Directly before using, the suspension of the nanocomposite was placed in an ultrasonic bath for 30 min in order to homogenize the dispersion.

### Preconcentration procedure

Two hundred-microliter aliquot of the suspension of Al_2_O_3_/nano-G (1 mg) was added into 25 mL of water sample. Then, the 0.1 mol L^−1^ HNO_3_ and 0.1 mol L^−1^ NH_3_aq were used to adjust the pH of the samples. Then, the sample was stirred for 5 min and passed through membrane filter in 2 min using the filtration assembly of 5-mm diameter. After drying for 5 min, the sample was measured by EDXRF.

The total time of sample preparation did not exceed 15 min. But using a multi-position magnetic stirrer and two vacuum filtration kits, a series of 10 samples in 30 min can be simultaneously prepared.

### Batch adsorption

The batch adsorption experiments were carried out with 1 mg of Al_2_O_3_/nano-G, 25 mL of Cr(III) solutions with specified concentration, and pH. The pH values of the suspensions of Al_2_O_3_/nano-G and Cr(III) were adjusted with HNO_3_ and NH_3_aq solutions. The suspensions were stirred for 90 min to achieve adsorption equilibrium. Next, the suspensions were filtrated through membrane filters. The amount of Cr(III) ions adsorbed on Al_2_O_3_/nano-G (mg g^−1^) was calculated from the difference between the initial concentration *C*_0_ (mg L^−1^), and equilibrium concentration *C*_e_ (mg L^−1^) determined in filtrate by the ICP-OES spectrometry: *q*_max_ *=* (*C*_0_ − *C*_e_)*V*/*m*_adsorbent_, where *V* is the volume of the suspension, and *m*_adsorbent_ is the mass of Al_2_O_3_/nano-G. The recovery is given as Recovery% = 100%(*C*_0_ − *C*_e_)/*C*_0_.

## Results and discussion

### Structural research

**Fig. 1:**
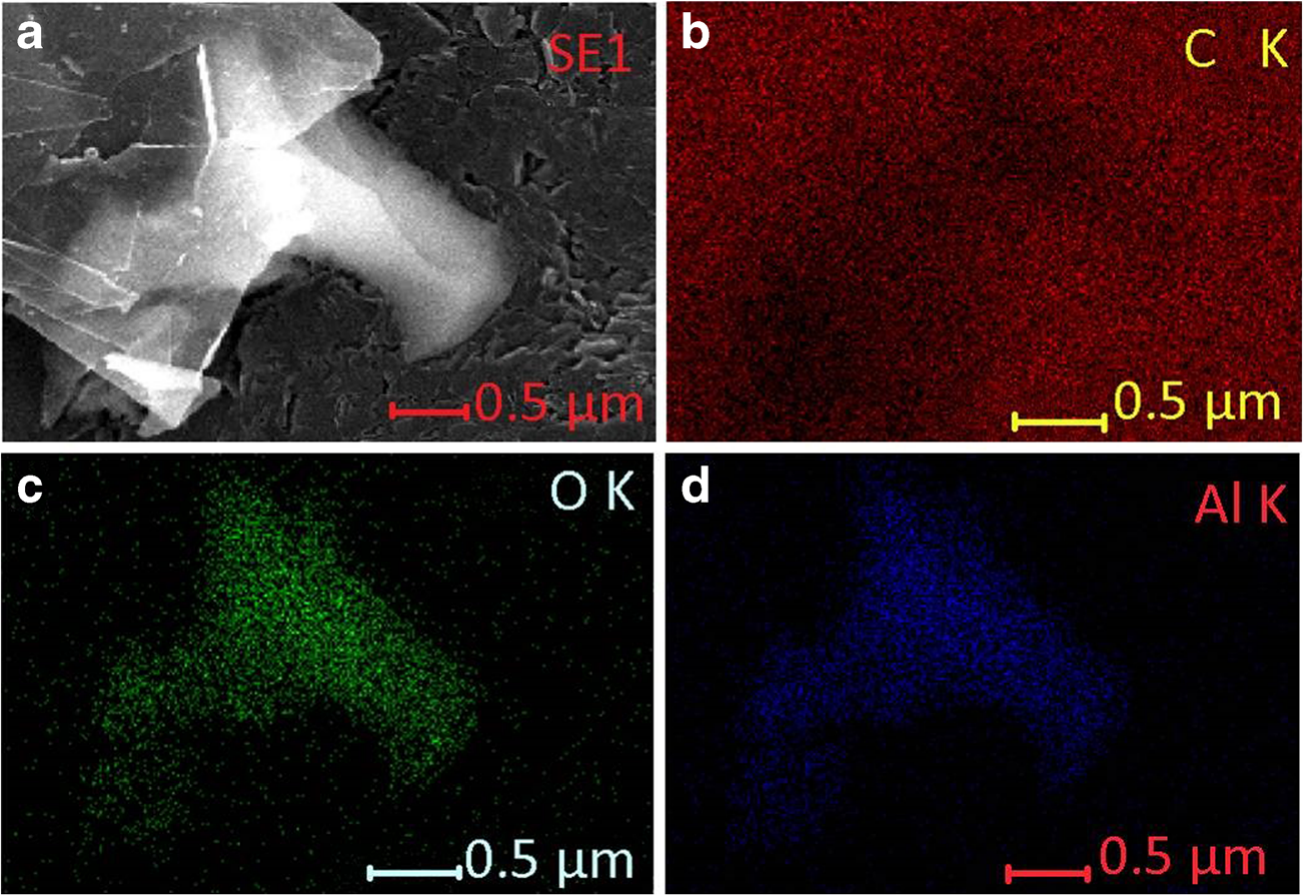
SEM images of synthesized Al_2_O_3_/nano-G (**a**) and maps of the correlation between distribution of carbon (**b**), oxygen (**c**), and aluminum (**d**) on the Al_2_O_3_/nano-G surface

The synthesized Al_2_O_3_/nano-G was characterized by SEM. Figure 1a shows a SEM image of Al_2_O_3_/nano-G composite. As can be seen, the alumina particles are shown on the surface of the thin layer of G nanosheets. The G nanosheets are semi-transparent suggesting their few-layer nature. Both the distribution maps of C, Al, and O elements (see Fig. 1b–d) in Al_2_O_3_/nano-G composite as well as the good correlation between distributions of aluminum and oxygen on the surface of nanocomposite confirm the presence of Al_2_O_3_ nanoparticles on the surface of nano-graphite.

**Fig. 2:**
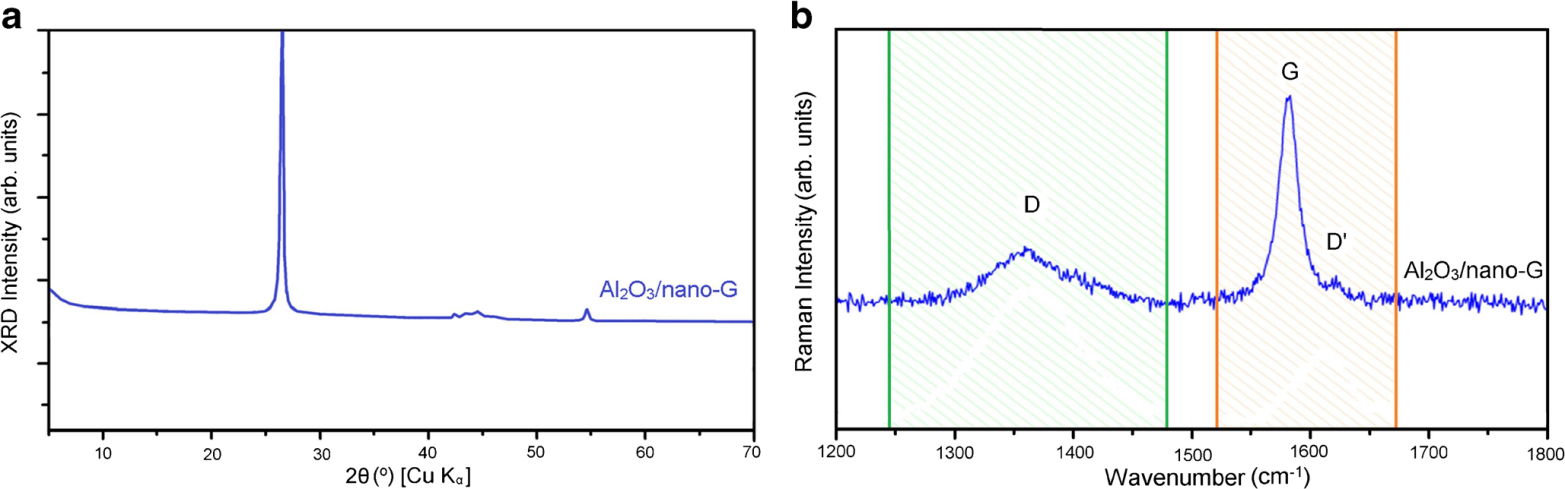
XRD patterns for Al_2_O_3_/nano-G (**a**) and Raman spectra obtained for Al_2_O_3_/nano-G (**b**)

Al_2_O_3_/nano-G was also characterized by XRD and Raman spectroscopy. Figure 2a presents XRD pattern of Al_2_O_3_/nano-G. The pattern for Al_2_O_3_/nano-G is characteristic of graphite; there are not any traces of crystallized Al_2_O_3_. The most pronounced graphite peak at 2*Θ* = 26.5° corresponds to coherently scattering hexagonal carbon layers with a *d*_002_ spacing of 3.36 Å. The diffractogram does not contain discrete patterns which could expose the presence of crystalline entities, including Al_2_O_3_. The absence of peaks for Al_2_O_3_ phase is due to the amorphous texture [26]. Instead, a plain background is recorded, with high low-angle intensity decreasing at higher theta angles. It resembles simulated XRD patterns for nano-sized, single layers of carbon structures [27].

The Raman spectrum of Al_2_O_3_/nano-G (see Fig. 2b) is characteristic of the nano-graphite. It consists of the intense G peak, at around 1582 cm^−1^, that originates from the *E*_2g_ vibrational mode of ordered in-plane sp^2^ carbons, and two, broad, and much less intense D and D′ bands at 1358 and 1621 cm^−1^, related to the disorder of edge carbons [28]. The intensity ratio *I*_D_/*I*_G_ is a measure of the disorder [29]. In Al_2_O_3_/nano-G, the *I*_D_/*I*_G_ is equal to 0.51 indicating the prominent disorder of the G nanosheets. The main source of the disorder comes from the small size of the G nanosheets, as it is indicated by the XRD and the presence of the oxygen functional groups as well as the number of areas of sp^2^ carbons with an alternating pattern of single-double carbon bonds [30] that generate huge blue shift of the G band to 1613 cm^−1^. The absence of peaks related to Al_2_O_3_ confirms its amorphous structure.

### pH effect

**Fig. 3:**
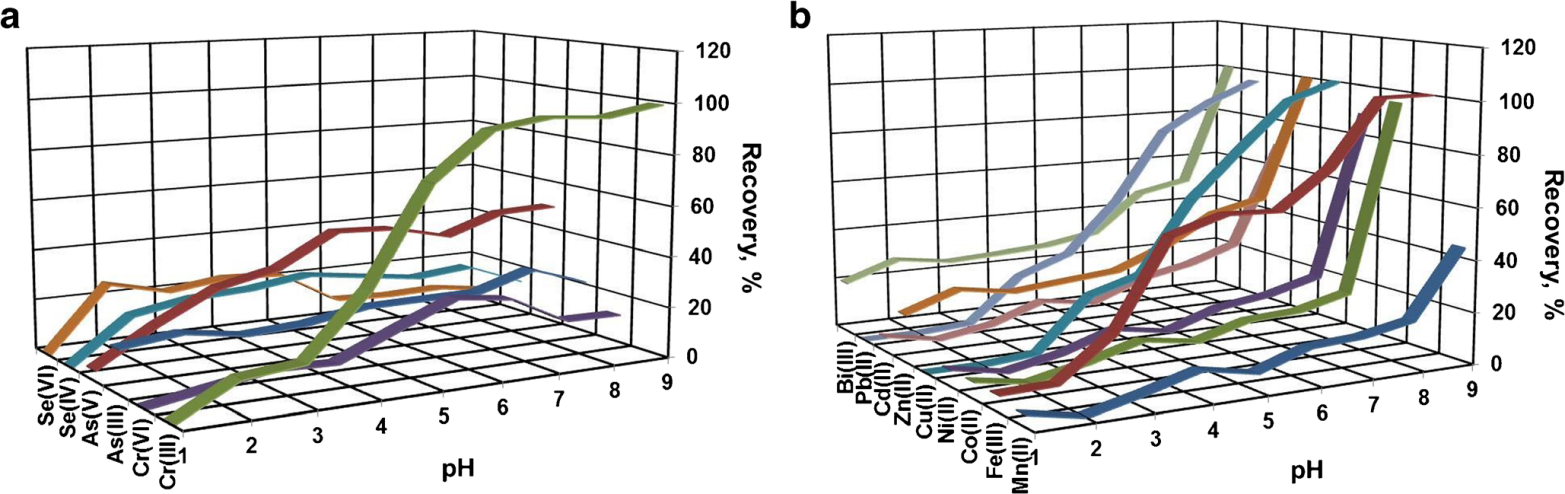
pH effect on adsorption of many metal ions including heavy-metal ions on Al_2_O_3_/nano-G; Cr, As, and Se (**a**); Mn, Fe, Co, Ni, Cu, Zn, Cd, Pb, and Bi (**b**) (working conditions: 1 mg of Al_2_O_3_/nano-G; *V* = 25 mL; *T* = 25 °C; *C*_analyte_ = 250 ng mL^−1^; *t* = 90 min; finally technique ICP-OES)

The effect of pH of aqueous solutions was investigated between 1.0 and 9.0 for a wide range of metal ions including heavy metal ions. Figure 3 presents relationship between adsorption percentage of multivalent ions, such as Cr(III), Cr(VI), As(III), As(V), Se(IV), and Se(VI) (Fig. 3a) as well as mainly divalent ions, such as Mn (II), Ni(II), Cd(II), Pb(II), Co(II), Cu(II), and Zn(II) and also trivalent ions: Fe(III), Bi(III) (Fig. 3b), and solution pH. As can be seen, Al_2_O_3_/nano-G shows selective and high affinity for Cr(III) ions. In the acid solution up to pH 3.0, the adsorption percentage is near 20%. Between pH 4.0 and pH 6.5, the adsorption percentage increases and, at pH 6.5, Al_2_O_3_/nano-G achieved the maximum adsorption (95%). Above pH 6.5 and in the basic solution, adsorption percentage of Cr(III) remains at the high level. In the case of Cr(VI) ions, Al_2_O_3_/nano-G shows the maximum adsorption percentage (25%) in the range of pH 6.0–7.0. At pH < 6.0 and pH > 7.0, Cr(VI) ions are poorly adsorbed by Al_2_O_3_/nano-G with adsorption percentage below 20%. The following Cr(III) species in aqueous solution predominate: Cr^3+^ (pH ≤ 5.0), Cr(OH)_2_^+^ and CrOH^2+^ (pH ≅ 4.0–6.5), and Cr(OH)_3_ (pH > 7.0).

Other metal ions such as As(III), As(V), Se(IV), Se(VI), Mn(II), Fe(III), Ni(II), Cu(II), Zn(II), Cd(II), Pb(II), and Bi(III) are not quantitatively adsorbed by Al_2_O_3_/nano-G from the solution of pH < 8. Pb(II), Cu(II), and Fe(III) are adsorbed with satisfactory results (near 100%), but only in strong basic solution (pH ≥ 8). The adsorption percentages of Pb(II), Cu(II), and Fe(III) at pH 6 were ca. 55%, 55%, and 70%, respectively. The adsorption percentage for As(V) being about 40% in the range of pH 4.0–6.0 is not enough to preconcentration of trace amounts of these ions. On the other hand, sorption study at pH 9.0, where the adsorption percentage was the highest for Fe(III), Co(II), Ni(II), Cu(II), Zn(II), Cd(II), Pb(II), and Bi(III), cannot be recommended due to the precipitation of metal as, e.g., hydroxides. Finally, taking into consideration both the high adsorption of metal ions and the prevention of the precipitation of metal hydroxides, the subsequent experiments and analysis of a real sample were performed only for Cr(III) at a pH of 6.5.

### Adsorption capacity

The adsorption capacity of Al_2_O_3_/nano-G for Cr(III) ions was investigated using Langmuir [31, 32] and Freundlich [33] isotherms. Langmuir isotherm is described by *q*_max_—the maximum amount of metal ions adsorbed per unit weight of sorbent (Al_2_O_3_/nano-G) achieving at the high equilibrium ion concentration (mg g^−1^)—and *K*_L_—the constant related to the free energy of adsorption (L g^−1^). Freundlich isotherm is described by *K*_F_ and *n*, Freundlich constant related to the adsorption capacity (mg^1 − *n*^ L^*n*^ g^−1^), and the adsorption intensity, respectively.

$$ {q}_e=\frac{q_{\mathrm{max}}{K}_L{C}_e}{1+{K}_L{C}_e} $$

$$ {q}_e={K}_F{C}_e^{1/n} $$

**Fig. 4:**
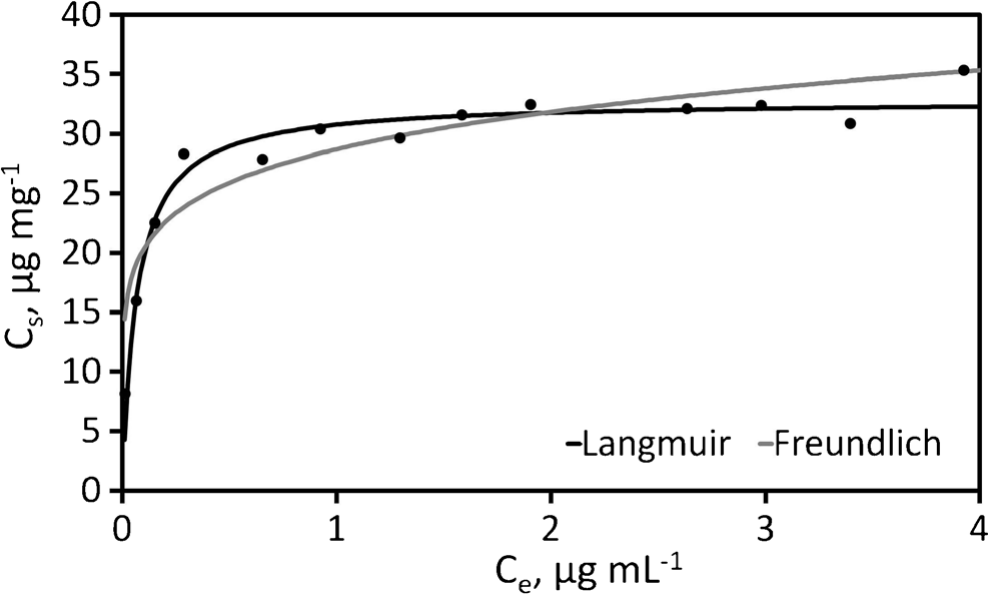
Langmuir and Freundlich isotherms for Al_2_O_3_/nano-G towards Cr(III) ions (working conditions: 1 mg of Al_2_O_3_/nano-G; *V* = 25 mL; *T* = 25 °C; *C*_0_ = 10 ng mL^−1^; *t* = 90 min)

Isotherm parameters were obtained by fitting the adsorption equilibrium data to the isotherm models. The following results for Langmuir and Freundlich isotherms are obtained: *q*_max_ = 32.8 mg g^−1^, *K*_L_ = 14.9 L g^−1^, *R* = 0.9339, *K*_F_ = 28.7 mg^1 – *n*^ L^*n*^ g^−1^, *n* = 6.68, *R* = 0.9123. Because the adsorption isotherms are fitted better by the Langmuir model than by the Freundlich model, a chemical adsorption process is suggested. Langmuir and Freundlich isotherms are presented in Fig. 4.

### Effect of contact time and sample volume

**Fig. 5:**
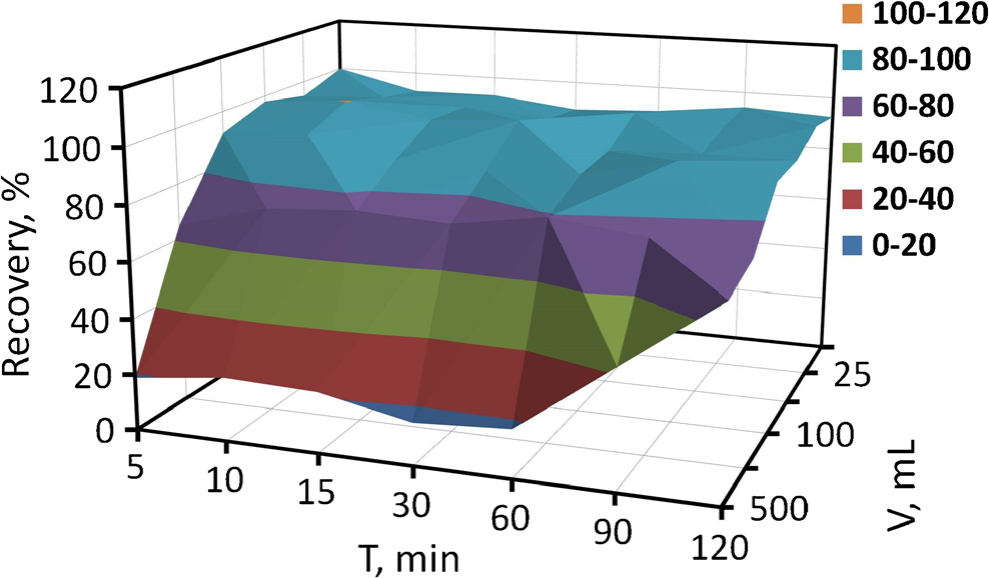
The effect of contact time combined with sample volume on the recovery Cr(III) (working conditions: ${\mathrm{m}}_{{\mathrm{Al}}_2{\mathrm{O}}_3/\mathrm{nano}-\mathrm{G}}$ = 1 mg; *C*_analyte_ = 10 ng mL^−1^; pH = 6.5)

The effect of contact time combined with sample volume on the adsorption recovery of Cr(III) ions on Al_2_O_3_/nano-G was studied in the range of 5–120 min and 10–500 mL, respectively. The obtained results are presented in Fig. 5. In the range sample volume from 10 to 100 mL, Cr(III) ions were adsorbed with recovery above 90% in the whole studied range of time (5–120 min). For the samples of 250 mL and 500 mL, the recoveries went down step by step. For a sample of 250 mL volume, recoveries decreased from 75 to 65% depending on the sorption time for 120 min and 5 min, respectively. For 500 mL of sample volume, the average recovery for time 5–60 min was ca. 20%, but when the time of sorption was 90 min and 120 min, the recoveries increased to 40% and near 70%, respectively. Consequently, DMSPE with Al_2_O_3_/nano-G as a sorbent can be recommended for the samples of volume ≤ 100 mL allowing to perform the sorption within only 5 min.

**Fig. 6:**
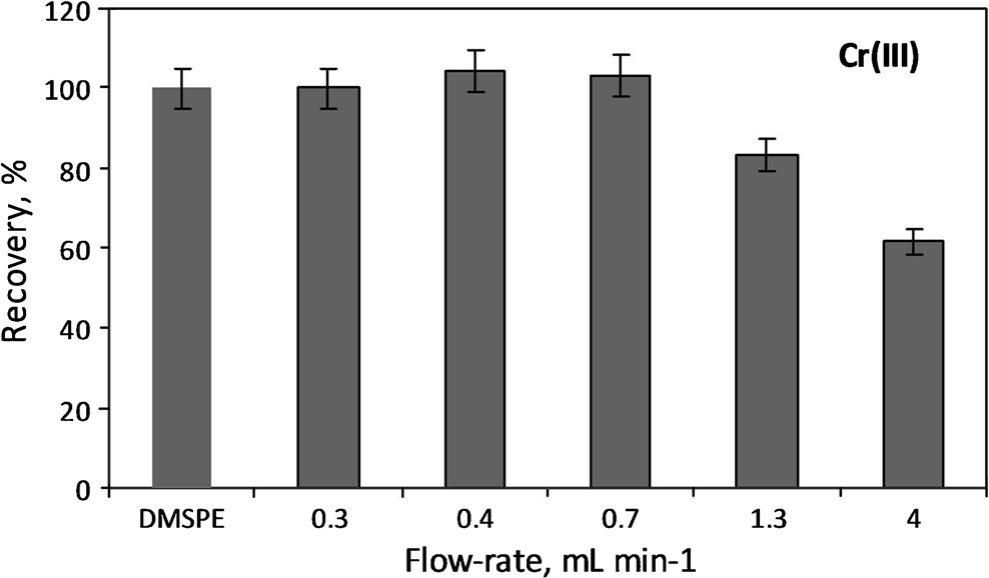
The effect of flow-rate for Cr(III) recovery (experimental details: Al_2_O_3_/nano-G membrane of mass per unit area of 0.32 mg cm^2^, *c*_analyte_ = 40 ng mL^−1^, pH = 6.5, *V* = 25 mL)

The Al_2_O_3_/nano-G composite is characterized by a large surface area and excellent dispersibility in water. Thus, it guarantees very good contact the nanocomposite with analyzed solution and in consequence the adsorption of ions as well as equilibrium state is achieved very fast (in less than 5 min as shown in Fig. 5). For comparison, the adsorption of Cr(III) ions under flow conditions was also investigated. For this purpose, the Al_2_O_3_/nano-G composite was deposited on filters via vacuum filtration, and then the analyzed solution was passed through loaded filters. The effect of flow-rate on chromium recovery is shown in Fig. 6. The results indicate that the adsorption of Cr(III) ions reaches the maximum value of 100% when flow rate is at least 0.7 mL min^−1^. So, it means that in order to obtain the high values of recovery, the adsorption of Cr(III) under flow conditions needs at least 35 min, i.e., five times longer than in the case of DMSPE (5 min).

### Effect of coexisting ions

The influence of coexisting ions on adsorption of Cr(III) on Al_2_O_3_/nano-G was investigated in the range of 20–200 μg mL^−1^ for K^+^, Na^+^, Ca^2+^, and Mg^2+^; 0.1–10 μg mL^−1^ for Fe^3+^; 100–800 μg mL^−1^ for NO_3_^−^; 0.1–1.0 μg mL^−1^ for PO_4_^3−^; 100–300 μg mL^−1^ for SO_4_^2−^; 50–400 μg mL^−1^ for Cl^−^; and 0.5–5.0 μg mL^−1^ for humic acid (HA). The results of these investigations are included in Table 1. The maximum studied concentration of Na^+^, K^+^, Mg^2+^, Ca^2+^, NO_3_^−^, PO_4_^3−^, and SO_4_^2−^ ions did not negatively affect the adsorption process of Cr(III) onto Al_2_O_3_/nano-G. According to WHO [34, 35] and EPA [36] regulations, the maximum concentrations of NO_3_^−^, SO_4_^2−^, and Cl^−^ in surface waters are in the range 0–18 μg mL^−1^, 0–250 μg mL^−1^, and 40–63 μg mL^−1^, respectively. Tolerance limit of Al_2_O_3_/nano-G in preconcentration of Cr(III) ions towards abovementioned ions are 45, 1.2, and 3.2 times better. In the groundwater, the concentrations of Mg^2+^, Ca^2+^ [37], Fe^3+^, NO_3_^−^ [36], SO_4_^2−^ [38], PO_4_^3−^, and Cl^−^ [36] are not higher than 100, 100, 2.0, 50, 230, 0.7, and 250 μg mL ^−1^, respectively. Thus, as obtained results show, nanocomposite can be also used in the analysis of groundwater. Cr(III) ions can be also successfully preconcentrated on Al_2_O_3_/nano-G using DMSPE when the concentrations of Fe^3+^ and HA are 2.5 μg mL^−1^ and 3.0 μg mL^−1^, respectively. Concentrations of iron in drinking water are normally less than 0.3 μg mL^−1^. Big tolerance of the proposed method towards coexisting ions allows applying Al_2_O_3_/nano-G as a sorbent to determination of chromium in different kinds of water such as ca. drinking and surface water as well as groundwater.

**Table 1:** Effect of coexisting ions and HA on adsorption process using Al_2_O_3_/nano-G as a sorbent in DMSPE/EDXRF procedure (experiment conditions: 1 mg of Al_2_O_3_/nano-G, 25 mL of sample volume, 10 ng mL^−1^ of Cr(III), pH = 6.5, adsorption time = 5 min)

Interferent	Concentration (μg mL^−1^)	Added as	Interferent/adsorbed ion ratio	Recovery (% ± RSD)
Na^+^	200	NaNO_3_	20,000	95.48 ± 0.65
K^+^	200	KNO_3_	20,000	105.36 ± 0.09
Mg^2+^	200	Mg(NO_3_)_2_·6H_2_O	20,000	95.61 ± 0.20
Ca^2+^	200	Ca(NO_3_)_2_·4H_2_O	20,000	94.65 ± 0.57
Fe^3+^	10	Fe(NO_3_)_3_∙9H_2_O	1000	75.67 ± 0.22
	5.0		500	82.18 ± 0.82
	2.5		250	96.75 ± 0.89
NO_3_^−^	800	KNO_3_	80,000	93.25 ± 1.14
PO_4_^3−^	1.0	Na_3_PO_4_·12H_2_O	100	91.98 ± 1.37
SO_4_^2−^	300	Na_2_SO_4_	30,000	100.16 ± 1.57
Cl^−^	400	NaCl	40,000	65.12 ± 1.08
	300		30,000	87.09 ± 0.42
	200		20,000	93.92 ± 0.48
HA	5.0	Humic Acid	500	80.62 ± 0.56
	4.0		400	85.07 ± 0.32
	3.0		300	94.73 ± 1.68

### Analytical performance

The calibration curve was prepared under optimized conditions based on series of standard solutions including various concentrations of Cr(III) from 2.0 to 50 ng mL^−1^. The characteristic of the method is presented in Table 2. Limit of detection (LOD) was calculated using the formula LOD = (3/*k*)(*B*/*t*)^1/2^, where *k* is the sensitivity read from equation (mg mL^−1^ s^−1^), *B* is the background count rate (counts s^−1^), and *t* is the counting time (300 s). Limit of quantification (LOQ) was calculated from LOQ = 3.3·LOD. The root mean square (RMS) describes the sum of the differences between the values of the standard and calculated concentrations. The method precision was characterized by average relative standard deviation (RSD).

**Table 2:** The parameters characterized DMSPE/EDXRF methodology (*n* = 3)

Analyte	Analytical range (ng mL^−1^)	Equation (*C*, ng mL^−1^) (*I*, count s^−1^)	Correlation coefficient (*R*)	LOD (ng mL^−1^)	LOQ (ng mL^−1^)	RMS (ng mL^−1^)	RSD (%)
Cr(III)	2.0–50	*I* = 4.943C + 1.146	0.9988	0.04	0.15	0.87	3.47

The proposed method can be applied to the simultaneous determination of Cr(III), Cr(VI), and total Cr. The recoveries were examined by spiking water samples with various Cr species. In order to determine total Cr in spiked water samples (see Table 3), the prereduction step using concentrated H_2_SO_4_ and ethanol was applied before using the developed methodology. Cr(III) was determined directly using the developed procedure. But the concentration of Cr(VI) was calculated by subtracting the concentration of Cr(III) from total chromium concentration. The results presented in Table 3 show that various species of chromium can be successfully determined in water samples by DMSPE/EDXRF with Al_2_O_3_/nano-G as a sorbent.

**Table 3:** Determination Cr(III) and Cr(VI) in spiked high-purity water

Added (ng mL^−1^)		Obtained (ng mL^−1^)		Recovery (%)	
Cr(III)	Cr(VI)	Cr(III)	Cr(VI)	Cr(III)	Cr(VI)
0	0	< LOD	< LOD	–	–
10	0	9.22 ± 0.68	< LOD	92.2	–
0	10	< LOD	9.51 ± 0.14	–	95.1
10	10	9.65 ± 1.12	9.38 ± 0.90	96.5	93.8

The developed method was also successfully applied for the determination of trace amounts of chromium ions in different types of drinking water samples, e.g., mineral and tap water. Analyzed water samples were spiked with 7.5 ng mL^−1^ and 15 ng mL^−1^ of Cr(III) ions. Table 4 presents the obtained results showing that the recoveries of enriched chromium (91–107%) are reasonable for trace analysis.

**Table 4:** Determination of Cr(III) in spiked water samples (pH = 6.5, sample volume 25 mL, 1 mg of Al_2_O_3_/nano-G, 5 min of contact time); *n* = 3; uncertainties correspond to one standard deviation

Sample	Added (ng mL^−1^)	Cr(III)	Cr(III)
		Obtained (ng mL^−1^)	Recovery (%)
High-purity water	0	< LOD	–
	7.5	6.94 ± 0.04	92.5
	15	13.73 ± 0.73	91.5
Mineral water^a^	0	< LOD	–
	7.5	7.93 ± 0.51	105.7
	15	16.01 ± 1.49	106.7
Mineral water^b^	0	< LOD	–
	7.5	7.68 ± 1.00	102.4
	15	15.86 ± 0.93	105.7
Tap water	0	1.25 ± 0.54	–
	7.5	8.33± 1.09	94.4
	15	17.3 ± 1.31	107.0

^a^Matrix, 5.00 mg L^−1^ (Na^+^); 1.35 mg L^−1^ (K^+^); 46.09 mg L^−1^ (Ca^2+^); 8.51 mg L^−1^ (Mg^2+^); 0.25 mg L^−1^ (F^−^); 5.30 mg L^−1^ (Cl^−^); and 172.68 mg L^−1^ (HCO_3_^−^). ^b^Matrix, 9.65 mg L^−1^ (Na^+^); 41.69 mg L^−1^ (Ca^2+^); 5.62 mg L^−1^ (Mg^2+^); 0.07 mg L^−1^ (F^−^); and 131.06 mg L^−1^ (HCO_3_^−^)

### Analytical application of Al_2_O_3_/nano-G

**Fig. 7:**
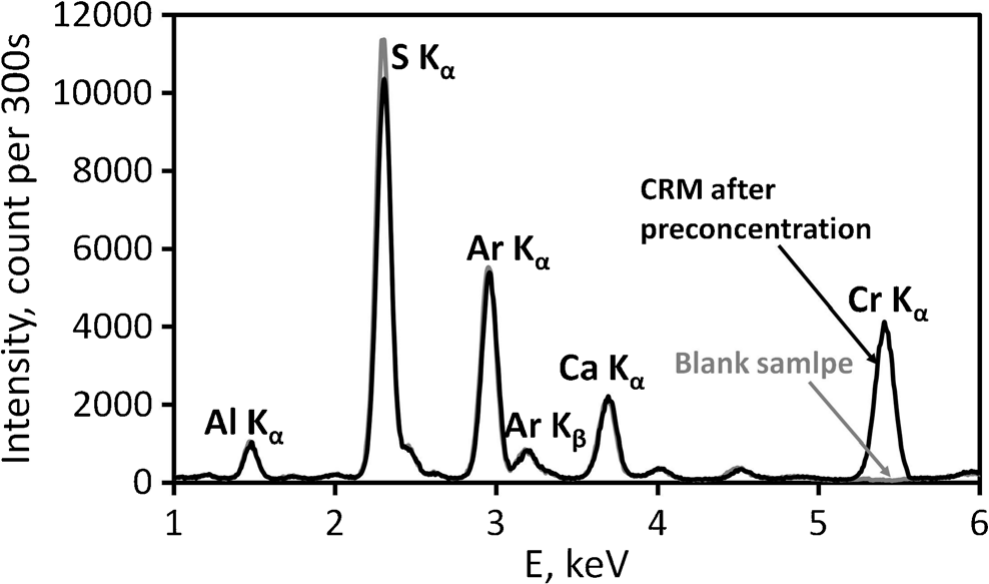
The EDXRF spectra for blank sample (gray line) and sample after reduction using H_2_SO_4_/C_2_H_5_OH and preconcentration of Cr ions (black line) in CRM on Al_2_O_3_/nano-G (measurement conditions: 20 kV, 450 μA, filter: Al-200, air)

The obtained Al_2_O_3_/nano-G composite combined with the developed DMSPE/EDXRF methodology was applied to analysis of certified reference material (CRM) of natural water NIST 1640a. The results are shown in Table 5. As can be seen, the accuracy of the method is very good, and the calculated error was below 3%. EDXRF spectra (see Fig. 7) confirmed good peak/background ratio for chromium after its preconcentration on Al_2_O_3_/nano-G, which results from the excellent sensitivity of the proposed methodology.

**Table 5:** Determination of chromium ions in CRM (results are expressed as mean values ± standard deviations, *n* = 3)

Water	Mayor element of matrix (mg L^−1^)	Major trace element of matrix (μg L^−1^)	Analyte	Certified concentration (μg L^−1^)	Determined concentration (μg L^−1^)	Error (%)
NIST 1640a	Ca (5.615 ± 0.021), Mg (1.0586 ± 0.0041), K (0.5799 ± 0.0023), Si (5.210 ± 0.021), Na (3.137 ± 0.031)	Al (53.0 ± 1.8), As (8.075 ± 0.070), Ba (151.80 ± 0.83), B (303.1 ± 3.1), Cr(40.54 ± 0.30), Co (20.24 ± 0.24), Cu(85.75 ± 0.51), Fe (36.8 ± 1.8), Mn(40.39 ± 0.36), Mo (45.60 ± 0.61), Ni(25.32 ± 0.14), Pb (12.101 ± 0.050), Se(20.13 ± 0.17), Sr (126.03 ± 0.27), U(25.35 ± 0.27), V (15.05 ± 0.25), Zn(55.64 ± 0.35)	Cr	40.54 ± 0.30	39.38 ± 0.39	2.85

### Comparison of DMSPE/EDXRF using Al_2_O_3_/nano-G with other methods based on solid-phase extraction

In Table 6, recently published methodologies used for determination of trace amount of chromium ions were compared with the developed methodology. As can be noticed, the different solid-phase extraction procedures and its miniaturized derivatives play an important role in the preconcentration of trace and ultratrace amount of chromium. The advantage of the developed methodology is a fact that only 1 mg of Al_2_O_3_/nano-G is enough to preconcentrate trace amount of chromium ions. Meanwhile, according to Table 6, the method using Fe_3_O_4_@SiO_2_@CTS magnetic nanoparticles [39], Fe_3_O_4_/Al_2_O_3_ [40], Fe_3_O_4_@ZrO_2_ [41], and Nb_2_O_5_–SiO_2_ [42] required 20, 30, 50, and 100 times higher mass of sorbent than synthesized Al_2_O_3_/nano-G. Furthermore, the mass of Al_2_O_3_/nano-G is less than the mass of the most other carbon sorbents based on modified CNTs and GO. Obtained LOD (0.04 ng mL^−1^) using the developed method and EDXRF as a measurement technique is better than LOD obtained using FAAS (in the range 0.34–1.60 ng mL^−1^) [17, 42] and TXRF (3.00 and 2.00 ng mL^−1^ for aqueous samples) [16]. Al_2_O_3_/nano-G combined with DMSPE/EDXRF methodology allowed achieving similar LOD (0.04 ng mL^−1^) to Fe_3_O_4_@SiO_2_@CTS magnetic nanoparticles connected with MSPE/ICP-OES (0.02–0.03 ng mL^−1^) [39], oxidized SWCNTs combined with SPE/ICP-MS (0.01–0.02 ng mL^−1^) [43], and GO with DMSPE/EDXRF (0.06 ng mL^−1^) [11]. Preconcentration of chromium on Al_2_O_3_/nano-G allowed us to decrease the LOD of this element three times in relation to Al_2_O_3_/GO nanocomposite used as a sorbent [24].

**Table 6:** Comparison of the developed DMSPE/EDXRF methodology with the other recently published procedures based on solid-phase extraction and its miniaturized derivatives

Procedure	Sorbent	Analyte	Mass of sorbent (mg)	pH	Analytical range (ng mL^−1^)	Final technique	LOD (ng mL^−1^)	RSD (%)	Ref.
MMHSPE^a^	Fe_3_O_4_/Al_2_O_3_ NPs	Cr(III)	30	8.0	10–1000	FAAS	1.40	3.4	[40]
MSPE^b^	Fe_3_O_4_@ZrO_2_ NPs	Cr(III)	50	8.0	4.0–400	FAAS	0.69	2.1	[41]
SPE-FI^c^	Nb_2_O_5_–SiO_2_	Cr(III)	100	8.0	1.2–120	FAAS	0.34	4.6	[42]
MSPE	Fe_3_O_4_@SiO_2_@CTS^f^ magnetic NPs	Cr(III)	20	9.0	0.1–100	ICP-OES	0.02	4.8	[39]
		Cr_total_		6.0			0.03	5.6	
SPE	Oxidized SWCNTs	Cr(III)	20	3.0	0.1–100	ICP-MS	0.01	2.1	[43]
		Cr(VI)					0.02	4.0	
SPE	Oxidized MWCNTs	Cr(III)	2.0	4.0	5.0–200	FAAS	1.15	1.7	[14]
DMSPE	Aliquat 336^j^-MWCNTs	Cr(VI)	5.0	2.0	10–3000	TXRF	3.00	11.9	[16]
				7.5	10–500		2.00	9.5	
DMSPE	GO	Cr(III)	0.5	6.0	1.0–150	EDXRF	0.06	1.7	[11]
Dispersive magnetic SPE	mf-GO	Cr(III)	50	8.0	5.0–100	FAAS	1.60	3.4	[17]
		Cr(VI)		2.0			1.40	3.0	
SPE	G	Cr(III)	30	8.0	10–1000	FAAS	0.50	4.3	[18]
DMSPE	Al_2_O_3_/GO	Cr(III)	1.0	6.0	2.0–50	EDXRF	0.11	4.0	[24]
DMSPE	Al_2_O_3_/nano-G	Cr(III)	1.0	6.5	2.0–50	EDXRF	0.04	3.5	This work

^a^*MMHSPE*, magnetic mixed hemimicelles solid-phase extraction; ^b^*MSPE*, magnetic solid-phase extraction; ^c^*FI*, flow injection system; ^f^*CTS*, chitosan; ^j^*Aliquat 336*, the anionic exchanger tricaprylmethylammonium chloride

## Conclusion

This paper described a composite based on nano-graphite (G) covered by aluminum oxide (Al_2_O_3_) in selective sorption and determination of trace chromium ions in water samples using DMSPE. Al_2_O_3_/nano-G combined with the developed methodology played also an important role in speciation of chromium. Thanks to the combination of DMSPE with non-destructive EDXRF as a measurement technique, the chromium ions were determined fast, simple, and effectively in water samples. The advantage of DMSPE/EDXRF methodology is that the elution of analyte is not needed and the Al_2_O_3_/nano-G with adsorbed Cr(III) can be analyzed directly. Thus, the developed methodology is eco-friendly and in agreement with the principles of green analytical chemistry. Compared to Al_2_O_3_/GO nanocomposite, Al_2_O_3_/nano-G is characterized by a much better limit of detection. The LOD achieved by the developed DMSPE/EDXRF procedure (0.04 ng mL^−1^) is below the highest licit concentration of chromium ions in drinking water according to EPA regulations (100 ng mL^−1^ of total chromium) [4]. Thus, the method can be recommended to determine chromium ions in water. The accuracy of method (expressed as an error) using certificate material reference NIST 1640a was 3%. The developed method allows determining the trace amount of chromium ions in environmental water samples without unnecessary laboratory work extending time of the analytical procedure and generating additional error sources.
